# Would you exchange your soul for immortality?—existential meaning and afterlife beliefs predict mind upload approval

**DOI:** 10.3389/fpsyg.2023.1254846

**Published:** 2023-12-14

**Authors:** Michael Laakasuo, Jukka Sundvall, Kathryn Francis, Marianna Drosinou, Ivar Hannikainen, Anton Kunnari, Jussi Palomäki

**Affiliations:** ^1^Department of Psychology and Logopedics, Faculty of Medicine, Helsinki, Finland; ^2^Faculty of Social Sciences, Department of Social Research, University of Turku, Turku, Finland; ^3^Department of Digital Humanities, Cognitive Science, Faculty of Arts, University of Helsinki, Helsinki, Finland; ^4^School of Psychology, Keele University, Keele, United Kingdom; ^5^School of Psychology, Department of Philosophy, University of Granada, Granada, Spain; ^6^Health and Well-Being Promotion Unit, Finnish Institute for Health and Welfare, Helsinki, Finland

**Keywords:** mind upload, moral psychology of AI, moral judgment, meaning in life, terror management, multidimensional existential meaning scale, afterlife beliefs, existential mattering

## Abstract

Mind upload, or the digital copying of an individual brain and mind, could theoretically allow one to “live forever.” If such a technology became available, who would be most likely to approve of it or condemn it? Research has shown that fear of death positively predicts the moral approval of hypothetical mind upload technology, while religiosity may have the opposite effect. We build on these findings, drawing also from work on religiosity and existential mattering as predictors of perceived meaning in one’s life. In a cross-sectional study (*N* = 1,007), we show that existential mattering and afterlife beliefs are negatively associated with moral approval of mind upload technology: people who believe there is a soul or some form of afterlife and who also report a high level of existential mattering, are least likely to morally approve of mind upload technology. Indeed, mind uploading—if it ever becomes feasible—is a form of technology that would fundamentally redraw the existential boundaries of what it means to be human.

## Graphical abstract

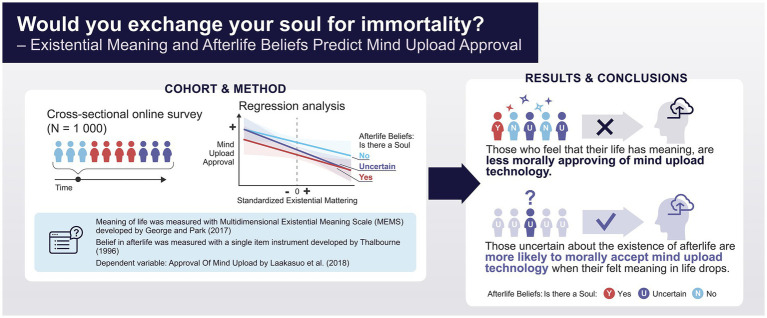

## Introduction

Mind upload refers to a speculative future technology that would allow the copying or transfer of an individual’s brain into a digital form (Moravec, 1988; Kurzweil, 2005; Wiley, 2014). While the current state of such technology is a “first draft” of a digital version of the connectome of a mouse neocortex (Reimann et al., 2019), some futurists predict that emulated human brains will become possible (e.g., Hanson, 2016). Realistic or not, the ethics and philosophy of mind upload have already been given serious thought (Sandberg, 2014; Cappuccio, 2017; Andrade, 2018). Here, we are interested in laypeople’s attitudes toward potential future technologies that could dramatically change humanity: who are the people who find mind upload moral or immoral, and why?

In terms of moral psychology, mind upload is especially interesting because it carries an implicit promise of immortality through technology, but in doing so, may clash with common intuitions about souls and the afterlife (Bering, 2006; Geraci, 2010; O'Connell, 2018). With respect to previous research, ours departs from common Terror Management Theory and Mortality Salience (Lifshin et al., 2016, 2018; Vail et al., 2020) research that has focused on comparing people’s literal beliefs in spiritual afterlife and people’s beliefs in symbolic afterlife (e.g., living in memories of conspecifics and artistic achievements). Previous research in psychology of life extension technologies has not focused on its moral psychological aspects. Nonetheless, one could consider mind-uploading as a literal form of secular afterlife and thus function with a different dynamic and logic than divine afterlife belief that buffers people against end-of-world beliefs in the form of, say, climate change.

Previous research has found both cultural and individual-level factors influencing people’s moral judgments about transhuman technologies (Laakasuo et al., 2018, 2021; Koverola et al., 2020). For example, exposure to science fiction increases acceptance of both cognition-enhancing brain implants and mind upload technology (Laakasuo et al., 2018; Koverola et al., 2020). Religiosity reduces approval of mind upload, though this was only observed in a sample from the United States and not in a sample from the more secular Finland (Laakasuo et al., 2018). Moral purity and sexual disgust sensitivity are associated with lower levels of acceptance, while Utilitarianism and Machiavellianism are associated with higher levels of moral acceptance toward mind upload (Laakasuo et al., 2018, 2021; Koverola et al., 2020). Death anxiety is associated with more positive moral judgments about mind upload (Laakasuo et al., 2018), suggesting that a fear of death may motivate acceptance, as mind upload seemingly allows one to live forever.

Here, we expand on what kinds of existential beliefs and attitudes motivate differing moral judgments about mind upload. Our research stemmed from the idea that mind upload can be argued to represent a secular version of immortality (Geraci, 2010; Lifshin et al., 2018; Vail et al., 2020), and from the aforementioned findings linking religiosity and death anxiety with moral disapproval and approval of mind upload, respectively. The present study focused on how beliefs about what happens *after* a person dies, coupled with the kind of value or meaning people feel in their own lives, affect one’s moral attitude toward a “technological afterlife.”

In a related stream of research (Lifshin et al., 2018), the authors describe how individuals high in religiosity rate medically achieved immortality as less plausible than those low in religiosity whose belief in indefinite life extension was mediated by their belief in souls. Vail et al. (2020) built on these results and showed that life extension manipulation reduced fear of death in atheists, whereas a supernatural immortality manipulation where a soul is said to live on in an afterlife did not. This implies that the possibility of life extension reduces atheists’ fear of death.

Prevailing theories in the psychology of religion have argued in favor of a bidirectional relationship between death anxiety and religious belief: namely, that death anxiety motivates religious belief (Vail et al., 2012), and religious belief in turn reduces death anxiety (see, e.g., Vail et al., 2010; Greenberg et al., 2020). However, the exact nature of this relationship is far from clear (Ellis and Wahab, 2013), and a recent review found evidence for the association to be weak or nonexistent (Jong, 2021). Van Tongeren (2019) argued that death anxiety and religiosity are linked, but the connection depends on the specific contents as well as the strength of religious belief (see also Wink and Scott, 2005), and the mediating role of meaning in life. Meaning in life (MIL) or existential meaning (George and Park, 2017) refers, in general, to the felt meaningfulness (being understandable, purposeful, consequential) of an individual’s life. Previous research suggests that more dogmatic religious beliefs are associated with stronger beliefs in an afterlife, which in turn are associated with stronger felt meaning in life (Van Tongeren et al., 2017). Greater felt meaning in life among religious individuals has been observed, for example, in participants’ open-ended responses to a writing prompt about their perceived sources of meaning in life (Nelson et al., 2021). Life meaning has been shown to mediate the attenuating effect of religiosity on death anxiety (van Tongeren et al., 2017), as well as its positive effects on broader psychological outcomes, including life satisfaction and self-reported well-being (Steger and Frazier, 2005). This proposed mechanism can help to explain why the association between religiosity and well-being has been found to be negligible (Chamberlain and Zika, 1988) when controlling for life meaning. Moreover, this mediating effect only affected those for whom religion was central to their identity (You and Lim, 2019). In sum, individual differences in afterlife beliefs and life’s felt meaningfulness seem to be important to the connection between religiosity and death anxiety, and thus, may also bring to bear on moral judgments about immortality technologies.^1^

Meaning in life has been historically divided into several different sub-dimensions with differing terminology, but recently a three-facet view has gained popularity (George and Park, 2016; Martela and Steger, 2016; Costin and Vignoles, 2020). Here, MIL is conceptualized as consisting of the distinct sub-constructs of comprehension, purpose, and mattering. Comprehension refers to a feeling of one’s life making sense, of things happening as they should; purpose refers to a feeling of one’s life having a direction and clear goals to move toward; and mattering refers to a feeling of one’s life, in its entirety, having significance and being consequential. Recently, Costin and Vignoles (2020) showed that individuals’ experienced existential mattering predicted their felt meaning in life, purpose, and comprehension. George and Park (2014) defined existential mattering “as the degree to which individuals feel that their existence is of significance and value; to feel a sense of EM is to feel that one’s existence is important and relevant.” The effect of existential mattering on felt meaning in life was not mediated by religion (whether participants identified as religious or not). Thus, while MIL is associated with religiosity, the most consistent predictor of MIL predicts it for atheists and religious people alike.

In the present study, we investigated whether views on the existence of souls, along with MIL, predict moral judgments of mind upload. Given previous findings linking death anxiety and religiosity to judgments about mind upload (Laakasuo et al., 2018), and the associations between religiosity and MIL (see Van Tongeren et al., 2017), we expected that lower MIL and not believing in an afterlife would be associated with higher approval of mind upload. Although meaning in life is associated with religiosity, it is also possible that feeling successful in one’s life is also purposeful and meaningful. The Terror Management Theory and Mortality Salience literature suggests that the symbolic immortality achieved by leaving a legacy that lives on after one dies can represent a type of immortality for *both* religious and non-religious individuals. It is thus possible that high score in meaning in life is indicative of investment in symbolic immortality. This would also indicate that those with less meaning in their lives and who do not have investment in a literal immortality belief in a soul can express greater interest in mind upload as an alternative form of immortality.

## Methods

### Participants and design

We recruited 1,040 participants on Prolific.com to participate in a correlational study. After exclusions, the final sample size was 1,007 (46% male; Age: *M* = 37.55, *SD* = 13.32; about 60% had at least a Bachelor’s degree or higher). The distribution of religious denominations in the sample is shown in Table 1.

**Table 1:** Distribution of religious belief.

Belief	Frequency	%
Agnostic	322	31
Atheist	354	34
Monotheist	249	24
Pantheist	21	2
Polytheist	16	2
Other	40	4
Rather not say	41	4

We excluded participants who (i) failed attention checks, (ii) stated that English was not their native language, and (iii) completed the study suspiciously fast (less than 900 s). The survey took approximately 40 min, and those who participated received 4 € in compensation. The data collection was completed as part of a previously published and preregistered study (doi:10.17605/OSF.IO/2V3FJ). However, the variables described in the present report were not part of this preregistration, and the reported analyses should be considered exploratory. The data presented here has not been previously published anywhere.

### Data availability statement

All analyses and data will be made available on figshare.com upon the publication of this article (10.6084/m9.figshare.24495682).

## Materials

### Multidimensional existential meaning scale

This scale was developed by George and Park (2017), based upon reviewing decades of previous work on meaning in life studies. The Likert scale has three sub-factors with five items each. The purpose sub-factor measures the extent of perceiving direction in one’s life and motivation toward achieving personally valued goals (e.g., “I have aims in my life that are worth striving for”). High purpose scores indicate a clear sense of goals or ends which one is striving toward. The comprehension sub-factor measures the extent of feeling a sense of coherence and understanding in one’s life (e.g., “I know what my life is about”). High comprehension scores indicate that one’s life makes sense, and that life’s components are clear and fit well together. The mattering sub-factor measures the extent of feeling that one’s personal existence is significant, valuable, and important for the rest of the world (e.g., “Even considering how big the universe is, I can say that my life matters”). High mattering scores indicate that one’s life feels consequential and of profound value. In our sample, Cronbach’s alphas were 0.91, 0.92, and 0.86, respectively. All items were scored from 1 (strongly disagree) to 7 (strongly agree).

### Belief in an afterlife

This is a single-item instrument developed by Thalbourne (1996), where one option (out of six) is chosen: (1) “What we think of as the ‘soul,’ or conscious personality of a person, ceases permanently when the body dies” [extinctionist]; (2) “After death, the ‘conscious personality’ continues for a while on a different plane and then is reincarnated into a new body on Earth elsewhere; this reincarnation process occurs over and over again, and may culminate in the individual being absorbed into a Universal Consciousness” [reincarnationist]; (3) “The ‘conscious personality’ survives the death of the body; it does not reincarnate into another body, but continues to exist forever; there may (or may not) be a day when the dead rise again from the grave” [immortalist]; (4) “The ‘conscious personality’ survives the death of the body and is indeed immortal; it may be reincarnated into another body, this process occurring over and over again; there may (or may not) be a ‘Resurrection of the Dead’” [eclectic]; (5) “The ‘conscious personality’ survives death of the body, but I’m completely unsure as to what happens to it after that” [other believer]; and (6) “I am completely uncertain as to what happens to the ‘conscious personality’ at the death of the physical body” [agnostic]. We collapsed categories 2–5, since they were all related to some form of belief in the soul or conscious personality surviving the death of the body, and treated categories 1 and 6 as separate categories. With this categorization, we ended up with three roughly equally sized categories, which we henceforth label as no afterlife beliefs (category 1; *N* = 298); uncertain about afterlife (category 6; *N* = 369); and believers in afterlife (categories 2–5; *N* = 340).

### Mind upload vignette

The vignette was adapted from the study of Laakasuo et al. (2018) and consisted of a science fiction story describing a researcher who engages in uploading of his mind. In the story, the researcher injects himself with very tiny nano-robots. The nano-robots then enter his brain through the bloodstream substituting his nerve cells one by one. After the neurons have been replaced, the brain’s functions are uploaded (copied) onto a computer. After every single brain cell has been uploaded, the nano-machines shut down and the researcher’s body collapses to the floor. He then wakes up inside the computer. This procedure has been labeled a *Moravec transfer procedure* (Moravec, 1988). However, this version is based on Eliezer Yudkowsky’s work^2^ (see Appendix for a full version of the vignette). We intentionally decided to focus on a scenario that describes the death of the corporeal body to avoid creating a situation in which participants could consider that there are two copies of the same mind.

### Dependent variable: moral approval of mind upload

The dependent variable had nine items (e.g., “The scientist acted in a morally correct way.”), four of which were reverse-coded (e.g., “The scientist’s decision was appalling.”). All items were rated on a Likert scale from 1 (not at all) to 7 (very much). The items exhibited a very high Cronbach’s alpha (0.93), indicating high internal consistency, and were averaged to create a moral approval measure for which higher scores indicated greater approval of the scientist’s decision to upload his mind. See Appendix for all items. This DV was developed by Laakasuo et al. (2018).

### Procedure

After consenting to participate, participants first filled in measures reported elsewhere (Laakasuo et al., 2021),^3^ along with the Multidimensional Existential Meaning Scale. Then, they proceeded to read the vignette describing a scientist successfully uploading his mind (see Appendix). After reading the vignette, participants gave their responses to a battery of Likert questions measuring their moral approval of the scientist’s actions. After this, participants filled in the Afterlife Beliefs scale and gave their responses on demographic questions. Finally, participants were debriefed and redirected back to Prolific.

## Results

Mind upload approval was negatively correlated with each MEMS sub-scale; higher scores in MEMS comprehension, purpose, and mattering were associated with lower scores in mind upload approval. Also, as expected, each of the MEMS sub-scales were positively intercorrelated (see Table 2 for details).

**Table 2:** Zero-order correlations between independent and dependent variable.

	MEMS purpose	MEMS mattering	Mind upload approval
MEMS comprehension	0.62	0.57	−0.09
MEMS purpose		0.53	−0.08
MEMS mattering			−0.19

All correlations statistically significant at *p* < 0.01.

We ran an ordinary least regression (OLS) analysis with the afterlife belief measure (non-believers, uncertain, and believers) as a categorical predictor and mind upload approval as the dependent variable. Those with no afterlife beliefs had the highest approval of mind upload (B = 4.58; 95% CI [4.41, 4.74]), followed by those uncertain about the afterlife (B = 4.21; 95% CI [4.07, 4.36]), and those with some form of afterlife beliefs had the lowest endorsement of mind upload technology (B = 3.91; 95% CI [3.75, 4.06]; all pairwise comparisons *ps* < 0.012).

Since the MEMS sub-scales were intercorrelated and associated with mind upload approval (see Table 2), we explored potential mediation effects between the variables. We ran a multiple OLS regression analysis by first entering all of the MEMS sub-scales as individual predictors (with mind upload approval as the DV) and thereafter as simultaneous predictors. In the separate analyses, each subscale had a significant negative association with mind upload approval (*B*_comprehension_ = −0.10, *t* = −3.1, *p* = 0.001; *B*_purpose_ = −0.09, *t* = −2.68, *p* = 0.007; *B*_mattering_ = −0.18, *t* = −6.45, *p* < 0.001). However, when entered as simultaneous predictors, the MEMS “mattering” subscale fully mediated the effects of both “comprehension” and “purpose” on mind upload approval [*B*_mattering_ = 0.20; *t*(1003) = −5.62, *p* < 0.001; with *B*_comprehension_ = 0.01, *t* (1003) = 0.3, *p* = 0.74; B_purpose_ = 0.02, *t*(1003) = 0.58, *p* = 0.55]. Thus, mattering seemed to be the primary driver of any effects of felt existential meaning on moral judgments about mind upload. This result aligned conceptually with finding of Costin and Vignoles (2020) that of the MEMS sub-scales, “mattering” was the most consistent predictor of other measures of meaning in life. Thus, the result may be interpreted in a simplified way as the effect of felt meaning in life on moral judgments.

**Figure 1:**
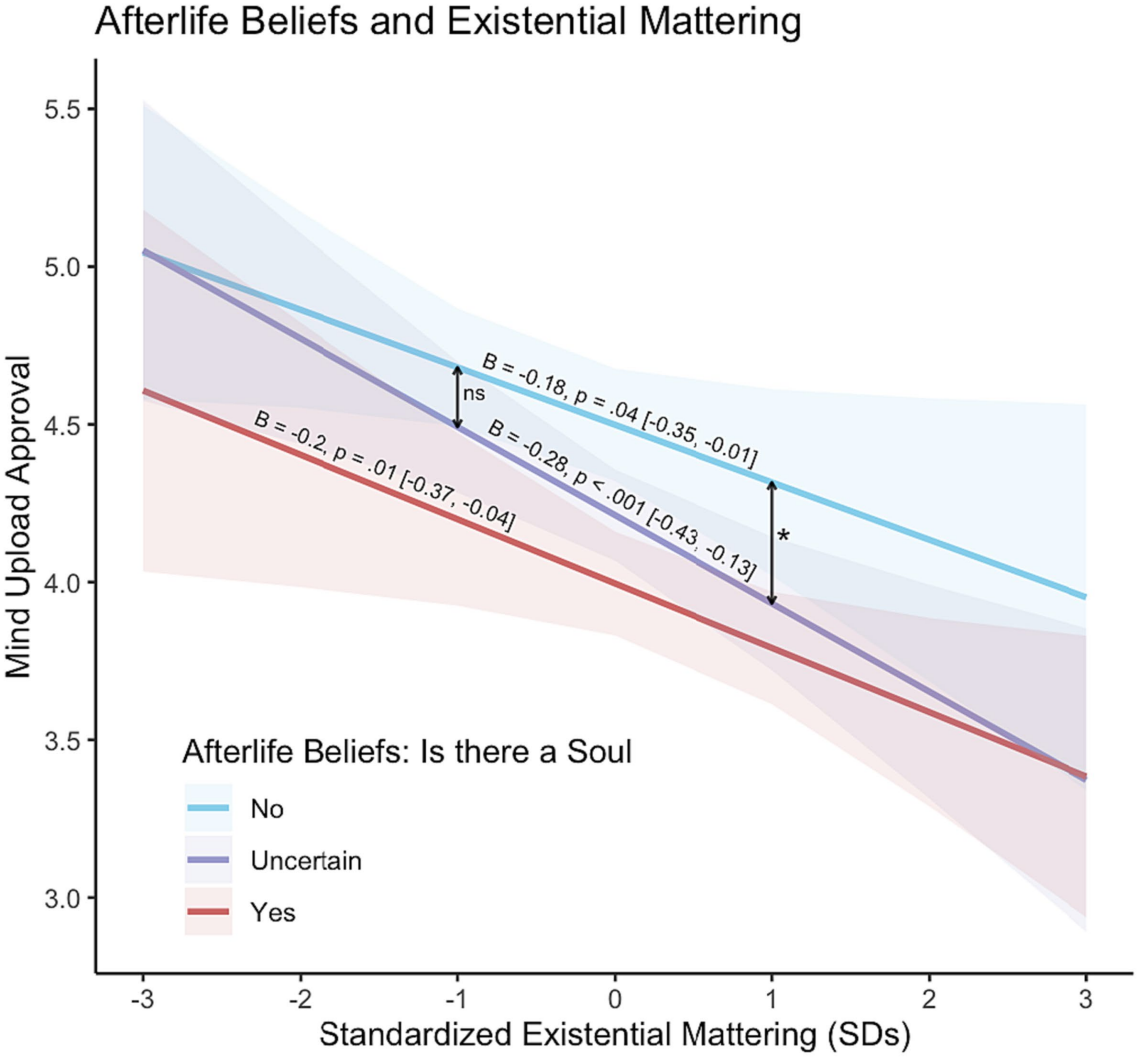
Moderation analysis between afterlife beliefs and the MEMS mattering sub-scale, predicting mind upload approval. There is a clear main effect for existential mattering, where an increase in felt mattering predicts lower levels of mind upload approval. The slope for those who are uncertain in their afterlife beliefs is the steepest.

Since religiosity and MIL are correlated, we sought to disentangle their independent effects on moral judgments in our results (focusing only on the Mattering subscale for simplicity). We first fit an OLS regression model with the MEMS Mattering subscale, the categorical afterlife beliefs (yes, no, and uncertain), and their interaction, as predictors, and mind upload approval as the DV. Modeling the interaction between Mattering and afterlife beliefs allows for estimating the simple effects of Mattering on mind upload approval for each afterlife beliefs category (see Figure 1, which visualizes the linear effects of Mattering on mind upload approval separately for the three levels of afterlife beliefs). Specifically, the effect of Mattering was strongest for those who were uncertain about the existence of an afterlife (B = −0.28; 95% CI [−0.43, −0.12]), followed by those who were certain that there is an afterlife (B = −0.20; 95% CI [−0.37, −0.04]) or certain that there is no afterlife (B = −0.18; 95% CI [−0.35, −0.01]). We also explored the differences between afterlife belief groups at low and high levels of mattering. At −1 SD of mattering, there was no difference between those who were certain there is no afterlife and those uncertain. At +1 SD of mattering, the uncertain group was significantly different from those who did not believe in afterlife (B = 0.39, 95% CI [0.01, 0.78], *p* = 0.04), but not different from those who did (see Figure 1).

To summarize, beliefs about the afterlife predicted moral approval of mind upload in a linear fashion, with those who were certain that there is an afterlife having the lowest approval, those who were certain that there is no afterlife having the highest approval, and the uncertain placing in the middle. Additionally, existential mattering negatively predicted moral approval of mind upload, while comprehension and purpose had only an indirect effect through mattering. Regardless of what the participants believed about what happens to a person after they die, an increase in mattering predicted a decrease in moral approval toward mind upload. Interestingly, the slope for those who were uncertain about afterlife was the steepest, and statistically significantly different from those who did not believe in an afterlife.

## Discussion

We investigated the associations between moral judgments about mind upload, afterlife beliefs, and existential meaning. Our results can be summarized as follows: First, individuals who did not believe in any form of afterlife approved of mind upload more than those who were certain that there is some form of afterlife. Second, individuals who were uncertain about whether there is an afterlife approved of mind upload more than those who were certain that there is an afterlife. Third, higher existential mattering—the belief that one’s life is in some way important in the grand scheme of the universe—was associated with lower approval of mind upload; this effect was pronounced for those who were uncertain about the afterlife. These findings complement previous work where mind upload approval was positively linked with death anxiety, science fiction hobbyism, Utilitarianism and Machiavellianism, and negatively with sexual disgust, religiosity, and moral purity (Laakasuo et al., 2018, 2021). More specifically, this study connects the mind-uploading research with Meaning in Life research and sets it in a dialogue with Terror Management Theory and Mortality Salience research (Lifshin et al., 2016, 2018; Vail et al., 2020). TMT research has introduced the distinction between symbolic immortality and literal supernatural and secular immortalities, and here we show that this perspective is fruitful in discussing mind uploading as well. The results here could indicate that when atheists have the option of a literal secular immortality, that buffers them against death anxiety similarly to those who have the option of supernatural afterlife available to them. Given that moral psychology of AI and transhumanism did not start TMT and MS themes in mind, it strengthens both research traditions when scientists independently find similar conclusions, although they walk different paths.

So, why are afterlife beliefs predictive of mind upload judgments? On first glance, it seems that people align their morality with their metaphysical worldviews. It makes sense that individuals who do not believe in existence after death are more approving of mind upload: a technological solution seemingly offering eternal life should appear positive to those who believe there is nothing after death (Lifshin et al., 2016, 2018; Vail et al., 2020). Correspondingly, if someone believes that life goes on after death, they have no reason to endorse a digital extension of biological life. An individual’s disapproval of mind upload can even be seen as *rational,* given their beliefs (see Stark, 1999 for rational choice theory of religious beliefs).

Compared to previous research, our study measured moral approval of the technology and not for instance agreement with scientific statements reports on the end of the world (e.g., Lifshin et al., 2016) or possibility of a an medically extended indefinite life (Lifshin et al., 2018). Judging an act as wrong or unacceptable is different from feeling that the act is unnecessary or measuring agreement on the potential realism of such technologies. That is, believers in an afterlife may feel no need for a technology that would “side-step” death, but such feelings do not necessarily imply less moral approval. Most people do not need to wear glasses, but moral condemnation of people wearing glasses is not common; indeed, such condemnation would seem strange to most people. It makes sense that non-believers in afterlife would find it desirable to have a technology that could make afterlife possible. This individual-level desire could be one of the factors that increases moral approval. However, this line of reasoning does not necessarily work symmetrically for those who do believe in an afterlife: they may not feel any desire to have access to mind upload technology, but a lack of desire is not equivalent to aversion.

Rather, it seems more likely that believers in an afterlife have other moral views associated with their worldview. One possible candidate is the condemnation of suicide, since religious individuals are less accepting of it (Stack, 2013). People may view mind upload as a kind of suicide: the person in the vignette is acting alone, engaging in an act that leaves their physical body showing no signs of life. More generally, both committing suicide and artificially extending one’s life may be seen by religious people as acts that violate a perceived natural order or God’s will. Based on findings that link suicide condemnation to specific beliefs about one’s life belonging to God (Ross and Kaplan, 1994; Worthen and Yeatts, 2001), Bering (2006, pp. 459) argued that the religious condemnation of suicide is essentially a judgment about “cheating” God; meaning, it goes against a supreme being’s right to decide about the lives of humans. This line of reasoning could be applied to life extension as well. Even if mind upload is not seen as suicide but as extending one’s lifespan, it seems clear that it conflicts with a worldview where the authority to decide about life does not belong to humans.^4^ Indeed, while the data for this study was being collected, Waytz and Young (2019) published a paper where they introduced the concept of “Aversion to Playing God.” The authors showed that many scientific advancements, even if they are beneficial, are met with moral condemnation, if they enhance human capacities beyond the obvious.

To turn back to the details of our results, one must ask: why do stronger feelings of existential mattering—but not purpose or comprehension—predict lower approval of mind upload? Notably, in our study, when participants were both uncertain about what happens after death and had low levels of felt mattering, they were not different in their approval of mind upload from those who did not believe in an afterlife. However, when uncertain individuals reported higher felt mattering, their approval of mind upload resembled that of participants who did believe in an afterlife. In other words, mattering seems to reduce the effect of afterlife uncertainty. One possible explanation for mattering uniquely predicting moral judgments is that the mattering sub-scale measures another kind of certainty. Whereas the purpose and comprehension sub-scales concern one’s feelings about their life in the current moment, the mattering sub-scale also concerns the future. That is, high mattering implies that a person is more likely to think that their life will have a legacy regardless of what happens after their death (i.e., symbolic immortality), making techno-immortality irrelevant. In other words, perhaps the MEMS Mattering sub-scale is an intentional proxy measure for felt sense of achieved symbolic immortality (*cf.* Lifshin et al., 2016).

However, according to Park and George (2016) and George and Park (2017), mattering is linked with spiritual beliefs and, at times, traditional religiosity. While we did not collect a general spirituality measure, we did measure afterlife beliefs, and found that mattering had a similar effect on moral judgment among people *with* and *without* beliefs in afterlife. Thus, any unobserved mediation effects of spirituality would have to be aspects of spirituality that are not related to afterlife beliefs. One potential candidate is the belief that one’s body has divine qualities, which is associated with mattering (George and Park, 2017)—which would contradict the MEMS mattering as a proxy for achieved symbolic immortality. Thus, the effect of mattering on moral judgments of mind upload could stem from perceiving mind upload as a violation of the natural order of the human body and mind (Waytz and Young, 2019). This aligns with findings from previous studies showing that moral purity (Graham et al., 2011) is associated with disapproval of mind upload (Laakasuo et al., 2018). Perhaps mattering is also associated with suicide acceptance separately from religious beliefs; or maybe mattering partially stems from an acceptance of mortality, and immortality technologies threaten it? Notwithstanding, this is a hypothesis for future studies on the topic.

Like all studies, this study also has some limitations. The practical limitations of such a study are the standard one in any survey-based questionnaire. There are demand characteristics and participants might try to help the researcher by providing the answers they think scientists want to hear. Notwithstanding the standard limitations, the more substantial or theoretical limitations are associated with the vignette that may not be assessing only moral acceptance of mind upload, but perhaps it is capturing attitudes toward suicide as well. However, this same vignette was used in an earlier study (by Laakasuo et al., 2018), where the participants’ attitudes toward suicide and death anxiety were held constant. Doing so did not dilute the effects of other variables of interest (e.g., disgust sensitivity and science fiction hobbyism). Nonetheless, perhaps future studies could investigate other forms of mind upload, in which the possibility of death is ruled out.

Recent studies on the moral psychology of transhumanistic technology have been primarily descriptive: they have revealed associations between variables but have not explained why these associations exist (Laakasuo et al., 2018, 2021; Koverola et al., 2020). Likewise, despite our current novel findings, we cannot fully explain the associations we observed and further work is needed. It seems, that our current era of rapid technological development and social upheavals is a fruitful moment in time to investigate these topics (Harari, 2017).

## Conclusion

Our results contribute to ongoing discussions on the moral implications of transhumanist technologies, which have the potential to shape our thoughts on what it is to be human. In our study, those most hesitant toward mind upload were also the most spiritually-minded, having not only afterlife beliefs, but also the strongest self-reported feeling that they matter as individuals in the grand scheme of the universe. For individuals who were uncertain about whether there is an afterlife, the feeling of existential mattering decreased moral acceptance of mind upload more sharply than for those who were certain about their beliefs.

In sum, people who have less reason to worry about dying, whether due to belief in an afterlife or due to the certainty that their life is consequential, are more likely to morally condemn mind upload. However, the specific reasons for these associations remain unclear. Future work should attempt to shed light on the specific psychological mechanisms that underlie opposition to (and approval of) technological immortality.

## Data availability statement

Data and script for the variables reported in this manuscript are available at: 10.6084/m9.figshare.24495682.

## Ethics statement

Ethical approval was not required for the studies involving humans because all local laws regarding research ethics were followed. The studies were conducted in accordance with the local legislation and institutional requirements. The participants provided their written informed consent to participate in this study.

## Author contributions

ML: Conceptualization, Data curation, Formal analysis, Funding acquisition, Investigation, Methodology, Project administration, Resources, Supervision, Writing – original draft, Writing – review & editing. JS: Writing – review & editing. KF: Writing – review & editing. MD: Writing – original draft, Writing – review & editing. IH: Writing – review & editing. AK: Data curation, Writing – review & editing. JP: Formal analysis, Methodology, Visualization, Writing – review & editing.

## Footnotes

^1^It may be that inconsistent results regarding the relationship between religiosity and a fear of death are due to measures of religiosity not tapping into the aspects of religiosity that are the most important to this link.

^2^http://yudkowsky.net/obsolete/singularity.html

^3^In this study, the authors ran a pre-registered structural equation model where they predicted moral approval of mind upload three-domain disgust scale, high conflict moral dilemmas and the dark triad.

^4^Other potential religious or spiritual reasons for being less morally approving of mind upload include the idea that mortality gives humans perspective; worries about what happens to the soul, often differentiated from the mind, in the mind upload scenario; and seeing a technological version of the afterlife as a threat to faith or something that undermines reasons to be religious.
