# Supplementary material for: Would you exchange your soul for immortality?—existential meaning and afterlife beliefs predict mind upload approval
Source: Front Psychol. 2023 Dec 14;14:1254846. doi: 10.3389/fpsyg.2023.1254846 (PMC10757642; doi:10.3389/fpsyg.2023.1254846)
Supplement: Supplementary file 1 [file Table_1.DOCX]

**Appendix A. The Vignette**

*On the next page is a story set in the future. Read the story through and try to immerse yourself in the story as well as possible - even if it is not relevant to your life. After reading the story, please answer the questions about the story.*

By the year 2050, research into both computing technology and the human brain has taken huge steps forward. One of the researchers in the field is Henry Willington. 42 years old, he used to be a professor at the neuroscience department of a major university before deciding to pursue more independent research. He has been fascinated by the brain ever since seeing a colorful illustration of it in a picture book he had as a child, and has spent most of his life learning more about it. Besides neuroscience, he also has a passion for computers, and spends much of his free time programming.

A particular idea that combines these two passions is the notion of transferring a human mind to run on a computer. Many people have speculated with the idea and done preliminary research into it, but so far nobody has managed to carry it out, or even seriously attempted it. However, as a result of his long studies and some unpublished research he conducted at the university, Henry believes he has managed to put all the necessary pieces together. He intends to be the first one to carry out such a transfer. Because it would take a long time to acquire the necessary permits for human experimentation, and because he is confident in the safety of his technique, he decides to demonstrate it by transferring his own mind.

After setting everything up, Henry sits down in his office chair, inserts an IV needle into his arm, and activates the program. The needle injects into his blood a swarm of tiny machines the size of a cell, which find their way into Henry’s brain. The machines start by studying one of Henry’s brain cells, and send a copy of their observations into the large computer in Henry’s office. The computer uses this information to create a simulated copy of the brain cell in its memory. Once the simulation is perfect, one of the machines replaces the original cell, using the information from the simulation to completely imitate the cell’s behavior and functions. The actual activity of the cell is now being calculated in the computer: the machine is just a transmitter, sending the computer information about the cell’s environment and receiving in return instructions for how to behave and what kinds of messages to send to the other cells.

The machines then slowly repeat this process for each cell, until the computer contains a complete simulation of Henry’s brain. Although the activity inside Henry’s skull might look like the real thing to an outside observer, the machines are just acting on the basis of instructions received from the computer, and all of the actual thinking has been transferred to the simulation running in the computer’s memory. Once this point is reached, the computer runs a series of final checks to verify that everything happened correctly, and then disconnects the machines. Henry’s body collapses to the floor, and he awakens inside the computer, the transfer is complete.

**Appendix B. DV scale**

1. How moral do you find the scientist's decision?

2. How acceptable was the scientist's decision?

3. How appealing was the scientist's decision?

4. The Scientist's decision was appalling.

5. Thinking about the scientist's decision makes me angry.

6. The Scientist should be punished for what he did.

7. The Scientist acted in a morally correct way.

8. The Scientist's action should not be allowed by the law.

9. There was nothing wrong with the scientist's action.
